# A Path-Length Efficient, Low-Overhead, Load-Balanced Routing Protocol for Maximum Network Lifetime in Wireless Sensor Networks with Holes †

**DOI:** 10.3390/s20092506

**Published:** 2020-04-28

**Authors:** Phi Le Nguyen, Thanh Hung Nguyen, Kien Nguyen

**Affiliations:** 1 School of Information and Communication Technology, Hanoi University of Science and Technology, 1 Dai Co Viet Road, Ha Noi 11615, Vietnam; lenp@soict.hust.edu.vn; 2 Graduate School of Engineering, Chiba University, 1-33, Yayoi-cho, Inage-ku, Chiba 263-8522, Japan; nguyen@chiba-u.jp

**Keywords:** wireless sensor network, hole bypassing, network lifetime maximization, forwarding

## Abstract

In wireless sensor networks (WSNs) with holes, designing efficient routing protocols, which prolong the network lifetime, is one of the most critical issues. To this end, this paper proposes a new geographic routing protocol for WSNs named the load **B**alanced and constant **S**tretch protocol for bypassing **M**ultiple **H**oles (i.e., BSMH). In contrast to the existing works in the literature, the design of BSMH simultaneously takes into account the three factors that impacted the network lifetime, including routing path length, control packet overhead, and load balance among the nodes. Moreover, BSMH aims at minimizing the routing path length and the control overhead, while maximizing the load balance. We theoretically prove the efficiency of BSMH and extensively evaluate BSMH against the state-of-the-art protocols. The evaluation results show that the proposed protocol outperforms the others in various investigated metrics, not only network lifetime, but also routing path stretch, load balance, and control overhead. Specifically, BSMH prolongs the network lifetime by 30% compared to the existing protocols while guaranteeing that the routing path stretch is under 1.3.

## 1. Introduction

Recent years have witnessed the emergence of wireless sensor networks (WSNs) which consist of many tiny sensor nodes equipped with the capabilities of sensing, processing and transmitting [1,2,3,4,5,6,7,8]. In a WSN, sensor nodes collect information about physical phenomena such as temperature, humidity, vibrations, etc. from the surrounding environment and transfer to the base stations for further processing. There are numerous types of WSN such as terrestrial WSNs, underground WSNs, underwater WSNs, multimedia WSNs, mobile WSNs [9]. Applications of WSNs have been widely used in various domains including military target tracking and surveillance [10], natural disaster relief [5,6,7,8,11], agricultural and environmental monitoring [12,13], biomedical health monitoring [14], etc. A WSN is composed of sensor nodes deployed over a region of interest to monitor and (or) control the physical environment. A sensor node typically includes four components. The first one is a sensing subsystem that consists of one or more sensors for data acquisition from the surrounding physical environment. The second component is a processing subsystem comprised of a memory for storing data and a controller to perform computation. The third and fourth one is a wireless communication component for data transmission and a power supply component, respectively. In WSNs, every sensor node usually needs to accomplish two demanding tasks simultaneously, sensing and communicating.

While the communication is mandatory, in many WSN applications such as battlefield surveillance or earthquake monitoring, the full sensing coverage is also essential. In the full-coverage WSN, the network can achieve its duty only if all sensor nodes can sense and report the sensed data. Hence, the death of even only one node may cause the network to operate unfunctionally. For example, the former WSN application requires sensors detecting and reporting all occurred events since an enemy can appear anywhere at any time. Therefore, all sensors need to operate functionally. In the latter, the death of a sensor locating at the critical region (e.g., the center of the earthquake) may reduce the forecast accuracy significantly. In other words, the network lifetime during which the network operates properly depends on the lifetime of the sensor node, which depletes the energy first. Therefore, maximizing the network lifetime, which is the minimum lifetime of sensor nodes, is one of the most critical problems in WSNs.

As sensor nodes are equipped with only limited capability and non-rechargeable batteries [12], conserving energy consumption is vital in prolonging the network lifetime. Moreover, due to the short-range communication nature of sensor nodes, data transfer in WSNs is usually needed to bypass multiples hop to reach a destination. Hence, designing energy-efficient routing protocols for WSNs has received intense research attention [15,16,17,18,19,20,21]. In the routing layer, energy conservation can be achieved by *shortening the routing path* and *reducing the overhead* caused by control packets. Moreover, as mentioned earlier, the death of even only one node may damage the network operation. Accordingly, *balancing traffic* over the network to extend the network lifetime is another crucial designing factor of routing protocol in WSNs.

In such WSNs, geographic routing [22,23] has been widely used because of its simplicity and efficiency resulting from its local and stateless nature. However, when subjected to routing holes (i.e., regions without sensor nodes that have communication capability), geographic routing suffers from the so-called local minimum phenomenon, where packets are stopped at the hole boundaries [24,25]. The traditional scheme (known as perimeter routing) for that phenomenon is forwarding packets along the hole perimeters [23,26,27,28]. However, this scheme suffers from two serious problems: routing path enlargement and data concentration around the hole boundaries. Recently, to bypass the problems, the common idea is forming a forbidden area around each hole. As a result, there have been proposed forbidden areas with certain shapes such as circle [29,30], ellipse [31], hexagon [32]. Although the simplicity of shaped regions can reduce the control overhead, a significant enlargement on the routing path may occur. That is caused by the possibly substantial difference between the forbidden area and the hole. A recent proposal deals with the routing path enlargement problem by describing the forbidden area as the exact polygon whose vertices are the nodes on the hole boundary [33,34]. However, this approach may incur a significant control overhead since the information needed to represent the forbidden area may be very large when holes become complicated. Moreover, due to the static nature of the forbidden areas and routing paths, all the existing protocols cannot solve the load imbalance problem thoroughly. Novel approaches using the dynamic forbidden area have been proposed in [35,36,37]. However, these approaches cannot deal with general cases where there are multiple holes in the networks and the source, and the destinations may stay inside the holes’ convex regions.

In this paper, we propose a load-Balanced and constant Stretch protocol for bypassing Multiple Holes (i.e., BSMH), which is a new geographic routing protocol for bypassing multiple holes in WSNs [38]. The unique feature of BSMH design is jointly considering three essential factors, which are minimizing the routing path length, minimizing control overhead, and maximizing load balance. We have theoretically proved BSMH’s efficiency and its satisfaction following the design patterns. Moreover, we have implemented and evaluated BSMH against state-of-the-art protocols using simulations. The evaluation results show that BSMH outperforms the others in all investigated metrics.

The remainder of the paper is organized as follows. Section 2 presents the related works. Section 3 describes our proposed protocol. In Section 4, we theoretically analyze the protocol performance. Section 5 introduces the performance evaluations using simulations. Finally, Section 6 concludes the paper.

## 2. Related Works

Due to the short-range wireless communication nature of the sensor nodes, data transmission in WSN is performed in a multi-hop paradigm. Therefore, routing becomes an important issue that attracts a lot of attention from the research community [15]. Inherent characteristics of WSN including limited energy supply, limited computation power of sensor nodes and the large scale of network topology pose many challenges to the design of routing protocol. To conserve energy consumption, the routing protocol should be made as simple as possible.

Geographic routing [22,23] which exploits the local geographical information at the GPS-equipped sensors is widely accepted for its simplicity and efficiency. Geographic routing algorithms typically assume: a) that each network node knows its own and its neighbors’ positions and b) that the source of a message knows the destination’s position. The simplest geographic routing protocol is the greedy forwarding where each node chooses the next hop to be the neighbor node with the most geographical advantage to the destination. Geographic routing works well in networks where the nodes are deployed densely. However, with the occurrence of void areas (which are also called routing holes), i.e., the regions where the nodes have died out and hence, no longer participated in the network communication, geographic routing suffers from the so-called local minimum problem where there is no neighbor which is closer to the destination than the current node [25].

To bypass a hole, traditional schemes appropriately switch between greedy and perimeter forwarding modes, in the latter of which the data packets are forwarded along the hole boundary. These proposals require a specific embedding of a planar graph (e.g., Gabriel Graph), a complicated procedure based on a restrictive assumption about the underlying graph (e.g., a unit-disk graph) [23,26,27,28,39]. Although these traditional approaches can alleviate the local minimum phenomenon, they face two critical problems. The first problem is the enlargement of routing paths and the second problem is the traffic concentration around the hole boundary [40,41].

To deal with these two problems, a new approach has been proposed which we call forbidden area approach. The main idea is to create a forbidden area around every hole from which all the packets are kept to stay away from. Typically, forbidden area is a static region covering the hole and with simple shapes such as circle [29,30], ellipse [31], hexagon [32], convex hull [33,34] In [29], Yu et al., proposed a routing scheme wherein the forbidden area is a circle covering the hole. First, the nodes on the boundary of the holes are identified using Boundhole algorithm [24] and a virtual circle that exactly covers the hole is formed. The information of the circle is disseminated to all nodes on the boundary of the hole. When a source node *S* wants to send a data packet to a destination node *D*, it first sends the packet along the line *SD* by geographic routing. The node on the boundary of the hole receiving the data packet informs the source node of the information of the virtual circle. Then, the source node calculates an anchor location which is the intersection of two tangent lines from *S* and *D* to the virtual circle. The source node forwards the data packet to the node which is closest to the anchor location by geographic routing and this node will forwards the data packet to the original destination. A routing scheme using virtual hexagon is proposed in [32]. Similar to [29], after identifying the boundary of a hole using the Boundhole algorithm, information of the center and the radius of the virtual circle which exactly covers the hole is calculated. This information is transferred to all nodes on the boundary of the hole and helps to make the routing decision. Instead of a virtual circle, in [31] a virtual ellipse covering the hole is calculated and the information of the ellipse is sent to all nodes inside the ellipse. Other works using the forbidden area approach can be found in [30,33,42]. all these approaches can reduce the data congestion on the hole boundary, but they may create a new congestion area around the hole cover, instead. Moreover, routing paths may be enlarged due to the difference between the hole and the hole cover. [43] is a rare protocol that uses a dynamic forbidden area that is circle-shaped. Although this protocol can alleviate the traffic congestion around the forbidden area, it still suffers from routing path enlargement problems.

In [44], Won et al. proposed a protocol that can guarantee that the route stretch is upper bound by $\Theta \left(c\right)$, where *c* is the path length of the shortest path. In this protocol, the forbidden area is the convex hull of the hole. The packets then are routed along the shortest path from the source to the destination that goes through the vertices of the convex hull. This work is followed in [34] to address the problem of routing between nodes inside the concave regions of the holes. In [34], the authors proposed to describe the hole by a polygon whose vertices are all the nodes staying on the hole boundary. The routing path then is determined by the visibility graph whose vertices are vertices of the holes. The upper bound stretch was proved to $\Theta \left(\frac{D}{\gamma }\right)$, where *D* is the diameter of the network and γ is the communication range of sensor nodes). Although these two protocols are the rare ones that can provide stretch upper bound, they still suffer from the same problem, i.e., traffic concentration around the forbidden area, as the other protocols described above. Moreover, in these two protocols, especially [34], the information needed to represent the forbidden area depends on the holes and it may be significantly large when the holes become complicated. Consequently, they may cause extra overhead in disseminating and storing information about the forbidden areas.

Recently, Huang et al. tried to improve the load balance by exploiting energy information in making decisions [45]. Specifically, before sending data packets, every source node sends two so-called burst packets towards the destination. The burst packets are hole-bypassing, one goes along the right-hand side and the other goes along the left-hand side of the holes. These packets collect information on two anchor lists along the routing path. Upon arriving at the destination, the burst packets, with the anchor lists embedded, are pushed back to the source. When a source node has a packet to send, it randomly chooses an anchor list and embeds the location of the anchors into the packet header. The packet then is forwarded towards the anchors gradually, where the next hop is chosen based on the location and the residual energy of the neighbors. In contrast to the other geographic routing protocols, this protocol requires nodes to periodically broadcast beacons to update the energy information.

Several approaches exploit heuristic methods to maintain the load balance between the nodes. Yu et al. [46] proposed a scheme to avoid the local minimum problem by identifying the nodes staying inside the concave areas and prevent them from participating in data delivery. Although these schemes can shorten the routing path, they continue to suffer from traffic congestion surrounding the hole. In [47], the next forwarder node is chosen based on a so-called forwarding factor. This factor is proportional with the residual energy and inversely proportional with the distance to the destination of the neighbors. Accordingly, neighbors with higher residual energy and shortest distance to the destination are more likely to be chosen. In [48], the next hop is chosen based on a self-election paradigm. Specifically, upon receiving a packet, all the neighbors of the sending node will start a waiting timer and wait for its timer to expires before broadcasting the packet. The waiting time is proportional to the destination. To alleviate the hole, all the nodes maintain a so-called eligible nodes table which consists of only 1-hop neighbor closer to the destination. The nodes can identify themselves as a node on the hole region if their eligible nodes table is empty. When a node which is further from the destination receives a packet from a hole node, it broadcast the packet to its neighbors, and this process is repeated until the packet arrives at a node closer to the base station. Another routing protocol addressing the local minimum problem was proposed by Petrioli et al. in [49]. In this protocol, each node is assigned a color from a predefined color list. Upon receiving a packet, the nodes with even colors search for the next forwarder that has positive advancement, while the nodes with odd colors search for the next forwarder that has negative advancement to the destination. The loop-freedom of route determination of Rainbow has been proved theoretically.

Although all these heuristic approaches can alleviate the data congestion on specific areas, they cannot provide any guarantee on the route stretch.

## 3. Proposed Protocol

We propose a hole-bypassing routing protocol which addresses at the same time the three designing factors described in Section 1, i.e., minimizing the routing path length, maximizing the load balance and minimizing the control overhead.

In the following, we first briefly describe the metrics related to the three design factors. The routing path length can be evaluated through the so-called routing path stretch. The routing path stretch of a packet is defined by the ratio of its hop count to that of the theoretical shortest path. The average routing path stretch of a routing protocol is the average value of routing path stretches of all the routing paths determined by the protocol. Another metric that also reflects the routing path length is the delay of packets which are the duration from when the packets are sent until they successfully arrive at the destination.

The load balance is evaluated by the maximum packet forwarding ratio which is the maximum ratio of the number of packets forwarded by a node to the total number of packets sent. In general, the smaller maximum packet forwarding ratio reflects a better load balance. Another metric related to the load balance is the network lifetime which is the time from when the network starts till the first node dies.

The control overhead is measured by the total amount of control packets, where control packets are defined as packets that are not data packets.

We aim at designing a distributed geographic routing protocol that generates dynamic routing paths with the stretch upper bounded by a constant. This constant can be controlled to be as small as 1+ϵ (ϵ is a predefined positive number, which we call the stretch factor). The variation of the routing paths ensures the load balance over the network.

### 3.1. Protocol Overview

**Figure 1:**
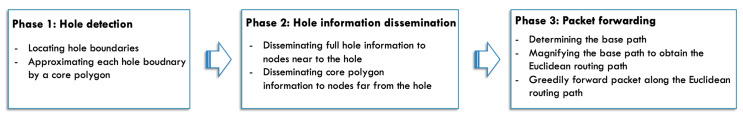
The overview of our approach.

We assume that the nodes do not know the hole location in advance. Therefore, before forwarding data, we need some setup phases that help to determine the hole as well as the core polygons and broadcast their information to the surrounding nodes. Our protocol consists of three phases as shown in Figure 1. The first phase is to detect the hole boundaries. For each hole, the boundary nodes determine a simple convex polygon covering the hole. This polygon is known as the *core polygon* of the hole. In the second phase, the information of the holes and the core polygons is disseminated to the nodes in the network. To reduce the overhead, we do not broadcast full information of all the holes to all nodes in the network. Instead, the broadcast information received by a node depends on the node’s location. Intuitively, nodes near a hole will receive the full information of the hole, while nodes staying far from the hole receive only the information of the hole’s core polygon. Once the first two phases finish, the nodes use hole information to make the routing decision. Specifically, for every packet, the source node first determines a base path to the destination. This base path is a Euclidean path that bypasses all the core polygons whose information stored in the local memory of the source node. The base path then is magnified using homothetic transformations to obtain the Euclidean routing path which will act as the guideline for the packet. The homothetic centers are chosen randomly to conserve the diversity of the routing paths, while the scale factors are controlled to guarantee the stretch upper bound. If the source node has full information about all the holes, then the packet is forwarded along the Euclidean routing path until reaching the destination. Otherwise, the packet is forwarded along the Euclidean routing path until arriving at an intermediate node which has more detail information about the holes. The intermediate node then redetermines the routing path to the destination and forwards the packet along the new routing path until reaching the destination.

We use the first two phases as in our previous work [37]. In the following, we first briefly describe the first two phases and then propose a new forwarding algorithm in the third phase.

### 3.2. Hole Determination and Hole Information Dissemination

All the nodes use TENT rule [24] to identify whether they are the stuck nodes (i.e., the nodes that may incur the local minimum phenomenon). Then, every stuck node initiates an HBA packet and sends this packet around its hole by using the RIGHT-HAND rule [24]. The HBA packets collect the coordinate of all the nodes it has traversed. Moreover, the boundary nodes also compute the core polygon covering the hole. Accordingly, when an HBA packet comes back to its initiator, it has the location of all nodes on the hole boundary as well as the coordinates of vertices of the core polygon. Please note that for the same hole, there may be multiple HBA created, thus to reduce the control overhead, we use an election algorithm to drop redundant HBA packet.

After the first phase, the initiator of the HBA packet has coordinates of all nodes on the hole boundary. This initiator creates a packet named HCI packet that conveys information of all hole boundary nodes and broadcast the HCI packets to the neighbors. When a node receives an HCI packet, it performs the following procedure:

- If the HCI packet contains information of a hole, then the node checks whether it already stored information of the hole in the local memory. If “yes”, it simply drops the HCI packet. Otherwise, it stores information about the hole into the local memory. Moreover, if the node has already stored information on the hole’s core polygon in the memory, then it removes the core polygon’s information because now it already has information on the hole. The node then checks whether it stay inside the hole’s vicinity. If “yes”, it broadcast the HCI packet. If “no”, if replace the information of the hole by the information of the core polygon before broadcast the HCI packet.
- If the HCI packet contains information of a core polygon, then the node checks whether it already stored information of the same core polygon or a hole whose core polygon is the core polygon in the HCI packet. If “yes”, the node simply drops the packet. Otherwise, the node stores information of the core polygon into the local memory and broadcast the HCI packet.

### 3.3. Data Forwarding Algorithm

The hardest challenge is how to reduce the traffic in the regions in between of the holes (we call this region the *critical region*). In [37], we proposed a data forwarding algorithm, in which the base path is always the shortest path bypassing the holes. However, in the context of networks with multiple holes, always use the same base path to construct the routing paths may cause a high traffic load in the critical region. To this end, we attempt to use dynamic base paths that vary for every packet. On the one hand, the base paths are probabilistically selected such that a path that is farther from the critical region is more likely to be chosen. On the other hand, the base path length is controlled to guarantee the required routing path upper bound. Specifically, the base paths are the Euclidean paths that bypass all the core polygons and have the length under a predetermined threshold. For each base path, we assign a so-called priority index which indicates how far is it from the holes. When a source has a packet to send, it probabilistically pickups one base path (with the probability being proportional with the path’s priority index) and scales up the base path by using homothetic transformations. The scale factors of the transformations are controlled to guarantee the required upper bound of the stretch and the scale centers of the transformations are chosen randomly to guarantee the diversity of routing path.

Let *s* and *t* be a source and a destination, respectively. In the following we will show how a source node *s* can exploit the hole information to make a routing decision to a destination *t*. To simplify the presentation, we use the following notations. Let *N* and *M* be two arbitrary nodes (N≠M), then C(N) denotes the core polygon containing *N*, and H(N) denotes the hole whose core polygon is C(N) (H(N)=C(N)=∅ if *N* does not stay inside any core polygon). Let $C(\bar{N})$ denote the set of all core polygons excepting C(N), and $C(\bar{N},\bar{M})$ denote the set of all core polygons excepting C(N) and C(M). Similarly, let us denote $H(\bar{N})$ the set of all holes excepting H(N), and $H(\bar{N},\bar{M})$ the set of all holes excepting H(N) and H(M). *N* is said a *M*-aware node, if either H(M)=∅ or *N* has the hole information of H(M).

**Figure 2:**
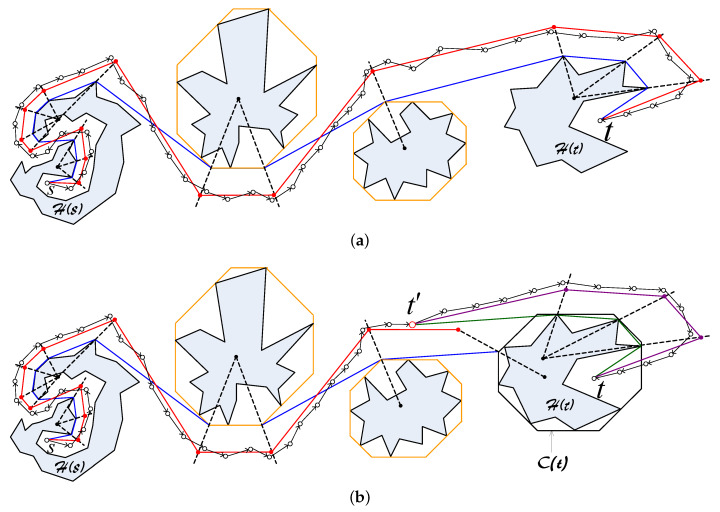
Data forwarding. The blue/green lines are the base path/sub-base path, the red/orange paths are the Euclidean routing path/sub-Euclidean routing path. The orange polygons are the items of $C(\bar{s},\bar{t})$. (**a**) Case 1: *s* is a *t*-aware node; (**b**) Case 2: *s* is a *t*-blind node.

Our data forwarding protocol consists of three steps: determining the base path, magnifying the base path to obtain the Euclidean routing path, and forwarding the packet along the Euclidean path. Before going to the detail of each step, we sketch the overview. If *s* is a *t*-aware node (i.e., *s* has information of the hole containing *t*), then *s* determines the base path as an Euclidean path from *s* to *t* which bypasses H(s), H(t) and $C(\bar{s},\bar{t})$. This base path then is magnified using a transformation to obtain the Euclidean routing path. Finally, the packet is forwarded along the Euclidean routing path until reaching *t*. See Figure 2a for the illustration.

Otherwise, if *s* is a *t*-blind node (Figure 2b) (i.e., *s* does not have full information of the hole containing *t*), H(t), thus it cannot determine a Euclidean path to *t* which bypasses all the holes. Therefore, *s* determines the base path as an Euclidean path from *s* to a vertex of C(t) which bypasses H(s) and $C(\bar{s})$. This base path then is magnified through a transformation to obtain the Euclidean routing path. The packet then is forwarded along the Euclidean routing path until reaching a node (let us call this node as $t^{\prime }$) that has the hole information of H(t). Please note that since the last vertex of the Euclidean routing path is a vertex of H(t), such a node $t^{\prime }$ always exists. With the hole information of H(t), $t^{\prime }$ determines a so-called *sub-base path* which is the shortest Euclidean path from $t^{\prime }$ to *t*, which bypasses $H(t^{{}^{\prime }})$, H(t) and $C(\bar{t},\bar{t^{{}^{\prime }}})$. This sub-base path then is magnified by a transformation to obtain a so-called *sub-Euclidean routing path*. The packet then is greedily forwarded along the sub-Euclidean routing path until reaching *t*.

In the following, we first present the algorithm to determine the base path in Section 3.3.1. Then, we describe how to magnify the base path to obtain the Euclidean routing path in Section 3.3.2.

#### 3.3.1. Determining the Base Path

We use dynamic base path that is probabilistically chosen from a set of base path candidates. Suppose *N* and *M* be two nodes in the network, we denote $X\left(N\right)=C\left(\bar{N},\bar{M}\right)\cup H\left(M\right)\cup H\left(N\right)$ if *N* is a *M*-aware node, and $X\left(N\right)=C\left(\bar{N}\right)\cup H\left(N\right)$, otherwise.

**Definition** **1** (Γ-bounded path). *An* Γ-*bounded path from N to M is a Euclidean paths bypassing all elements of X(N) and satisfying the following conditions:*

- *Its length does not exceed* Γ.
- The angle between any segment connecting its two consecutive vertices and vector $\vec{NM}$ is an obtuse angle.

*M* and *N* are said to be visible to each other if MN does not intersect the interior of $C(\bar{s},\bar{t})$, H(s) and H(t).

Below is the algorithm to determine the base path.

First, *s* constructs the visibility graph G=(V,E) as follows. If *s* is a *t*-aware node, then *V* consists of *s*, *t* and all the vertices of $C(\bar{s},\bar{t})$, H(t) and H(s). Otherwise, if *s* is a *t*-blind node, then *V* consists of *s*, and all the vertices of $C(\bar{s})$ and H(s). Two vertices of *V* is connected if they are visible to each other. *s* applies the breath-first search algorithm on graph *G* to determine the base path candidates as follows.

- If *s* is a *t*-aware node, the base path candidates are all paths from *s* to *t* that are either the shortest path or $\sin\frac{(n-2)\pi }{2n}\left(1+\epsilon \right)\left|L\left(s,t\right)\right|$-bounded paths, where L(s,t) is the shortest path from *s* to *t*, *n* is the minimum number of vertices of the core polygons.
- If *s* is a *t*-blind node, the base path candidates are all paths from *s* to a vertices on C(t) that are $\left(\Gamma_{1}\times \left|L\left(s,C(t)\right)\right|\right)$-bounded paths, where $\Gamma_{1}=Max\left\{1,\frac{\sin\frac{(n-2)\pi }{2n}\left(\left(1+\epsilon \right)d_{C(t)}\left(s\right)-\frac{p_{C(t)}}{2}\right)}{d_{C(t)}\left(s\right)+\frac{e_{C(t)}}{2}}\right\}$ and $L\left(s,C(t)\right)$ denotes the shortest length of the shortest paths from *s* to a vertex of C(t).

Second, all base path candidates are assigned priority indexes as follows. Let B be the set of all base path candidate and $B_{1},B_{2},\ldots .,B_{\left|B\right|}$ be the base path candidates. Denote $B_{1}^{i},\ldots ,B_{|B_{i}|}^{i}$ as all the vertices of $B_{i}$. Let P be the set of all core polygons and $P_{1},P_{2},\ldots .,P_{\left|P\right|}$ be the core polygons. Denote $P_{1}^{u},\ldots ,P_{|P_{u}|}^{u}$ as all the vertices of $P_{u}$. We define the margin of a base path $B_{i}$, denoted as $m_{B_{i}}$, by the minimum distance from a vertex of $B_{i}$ to an edge of a core polygon and the distance of a vertex of a core polygon to an edge of $B_{i}$. More specifically,

$$m_{B_{i}}=\underset{j=\bar{1,|B_{i}|};u=\bar{1,|P|};v=\bar{1,|P_{u}|}}{Min}\left\{d\left(B_{j}^{i},P_{v}^{u}P_{v+1}^{u}\right),d\left(P_{v}^{u},B_{j}^{i}B_{j+1}^{i}\right)\right\}$$

where d(A,l) depicts the Euclidean distance from a point *A* to a line segment *l*. The priority index of $B_{i}$ is defined as $1-\frac{m_{B_{i}}}{\sum_{i=1}^{\left|B\right|}m_{B_{i}}}$

Finally, *s* probabilistically chooses from the base path candidate set a base path such that the probability for a base path candidate to be chosen is proportional with its priority index. Algorithm 1 describes the pseudo code for determining the base path candidate.
**Algorithm 1:** Base path candidate determination algorithm.**Input ***s*: source, *d*: destination, ϵ: the stretch factor  **Output ***P*: *base path candidate set*
*P*;*shortest_len*← Shortest_path (*s*, *d*);**if** d_core*== null ||*d_hole*!= null*
**then**
$\Gamma_{1}=\frac{(n-2)\pi }{2n}\left(1+\epsilon \right)$ ;
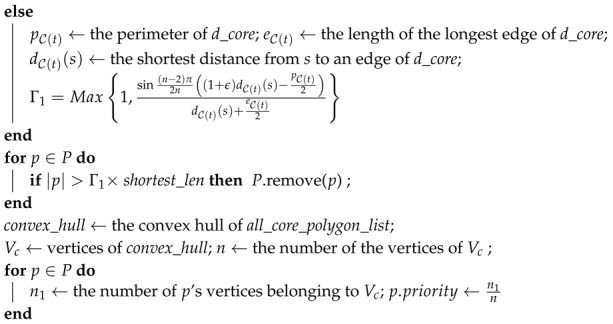
**return***P*

#### 3.3.2. Determining the Euclidean Routing Path

Let *N* and *M* be two arbitrary points on the plane, and P be a set of polygons on the plane. Let *l* be a P-bypassing broken line from *N* to *M*, which is comprised of boundary segments of P’s polygons. In the following, we define a transformation F(l,P,α) that transforms *l* to a new P-bypassing broken line $l^{\prime }$ whose length does not exceed α times that of *l*. α is call the scale factor of the transformation.

First, *l* is decomposed into segments, each of which is a boundary segment of a polygon of P. Then, for every segment that is a boundary segment of a concave polygon, it is further divided into sub-segments, each of which consists of consecutive edges of polygon staying on the same side with respect to the hole. Let us denote the segments/sub-segments composing *l* as $L_{1},\ldots ,L_{k}$. Then, each $L_{i}$ (i=1,…k) is scaled up using a specific homothetic transformation denoted as $F_{i}$, whose scale center (denoted as $O_{i}$), and scale factor (denoted as $\xi_{i}$) are defined as follows. Suppose $L_{i}$ consists of $n_{i}$ vertices ($n_{i}$ is a positive integer), and let us denote $L_{i}$’s vertices as $L_{i}^{1},\ldots ,L_{i}^{n_{i}}$. If $L_{i}$ is a boundary segment of a convex polygon, then $O_{i}$ is a random point staying inside the polygon whose boundary containing $L_{i}$. Otherwise, $O_{i}$ is a random point staying on the opposite side with the hole concerning the $L_{i}$.

(1) $$\xi_{i}=1+\left(\alpha -1\right)\frac{z_{i}+x_{i}}{x_{i}+y_{i}}$$

where $x_{i}=\left|L_{i}\right|$, $y_{i}=\left|O_{i}L_{i}^{1}\right|+\left|O_{i}L_{i}^{n_{i}}\right|$,

$$z_{i}=\left\{\begin{array}{c} \left|sL_{i}^{1}\right|+\frac{1}{2}\left|L_{i}^{n_{i}}L_{i+1}^{1}\right|,\;\mathrm{if}\;i=1\\ \frac{\left|L_{i}^{1}L_{i-1}^{n_{i-1}}\right|+\left|L_{i}^{n_{i}}L_{i+1}^{1}\right|}{2},\;\mathrm{if}\;i=\bar{2,k-1}\\ \left|L_{i}^{n_{i}}t\right|+\frac{1}{2}\left|L_{i-1}^{n_{i-1}}L_{i}^{1}\right|,\;\mathrm{if}\;i=k \end{array}\right.$$

Let us denote by $L_{i}^{{}^{\prime }}$ the image of $L_{i}$ through the homothetic transformation $F_{i}$, then $F\left(l,P,\alpha \right)={⋃}_{i=1}^{k}L_{i}^{{}^{\prime }}$. Please note that when $\xi_{i}$ is too large, $L_{i}^{{}^{\prime }}$ (i.e., the image of $L_{i}$ obtained by using $F_{i}$) may intersect P. In such cases, $\xi_{i}$ is gradually decreased by a factor of $\frac{3}{4}$ until $L_{i}^{{}^{\prime }}$ bypasses P.

Let us denote the base path from *s* to *t* as B(s), Now by using the transformation *F* defined above, *s* constructs the Euclidean routing path as the image of B(s) through $F(B\left(s\right),A\left(s\right),\alpha_{1})$, where A(s) and $\alpha_{1}$ are defined as follows.

$$A(s)=\left\{\begin{array}{c} \left\{H\left(s\right),H\left(t\right)\right\}\cup C\left(\bar{s},\bar{t}\right),\;\mathrm{if}\;s\;\mathrm{is}\;a\;t-\mathrm{aware}\;\mathrm{node}\\ \left\{H\left(s\right)\right\}\cup C\left(\bar{s}\right),\;\mathrm{otherwise} \end{array}\right.$$

$$\alpha_{1}=\left\{\begin{array}{c} \frac{1+\epsilon }{\theta_{1}}\sin\frac{(n-2)\pi }{2n},\;\mathrm{if}\;s\;\mathrm{is}\;a\;t-\mathrm{aware}\;\mathrm{node}\\ Max\left\{1,\frac{\sin\frac{(n-2)\pi }{2n}\left\{\frac{\left(1+\epsilon \right)L_{0}}{\theta_{2}}-\frac{p_{C(t)}}{2}\right\}}{\theta_{1}\left(L_{0}+\frac{e_{C(t)}}{2}\right)}\right\},\;\mathrm{otherwise} \end{array}\right.$$

where $p_{C(t)}$ is the perimeter of C(t); $L_{0}=Max\left\{\sin\frac{(n-2)\pi }{2n}\right.\left.\left|l_{C}\left(s,C\left(t\right)\right)\right|-\frac{e_{C(t)}}{2},\sin\frac{(n-2)\pi }{2n}\left|l_{C}\left(s,C\left(t\right)\right)\right|\right.$ + $\left.\left|l_{G}\right|-e_{C(t)}\right\}$, $l_{C}\left(s,C\left(t\right)\right)$ and $l_{G}$ are the shortest Euclidean paths from *s* and *t* to a vertex of C(t), respectively. $\theta_{1}$ can be seen as the ratio of the base path length to the shortest path length, which is defined as follows. Specifically, $\theta_{1}=\frac{\left|B(s)\right|}{|l_{C}\left(s,t\right)|}$, if *s* is a *t*-aware node; and $\theta_{1}=\frac{\left|B(s)\right|}{|l_{C}\left(s,C\left(t\right)\right)|}$, otherwise. Algorithm 2 describes the pseudo code for determining the Euclidean routing path.

#### 3.3.3. Forwarding the Data Packet

Denote by $sR_{1}\ldots R_{m}x$ the Euclidean routing path constructed by *s* using the algorithm described above, where *x* coincides with *t* if *s* is a *t*-aware node, and *x* is a vertex of C(t), otherwise. Then, the coordinates of $R_{1},\ldots ,R_{m},x$ are inserted into the packet header as virtual anchors that will guide the packet.

If *s* is a *t*-aware node, then the packet is forwarded toward the virtual anchors using greedy algorithm until reaching *t*.

Otherwise, the packet is forwarded toward the virtual anchors using greedy algorithm until reaching the first *t*-aware node, hereafter we call this node as *sub-destination* and denote as $t^{\prime }$. Then, $t^{\prime }$ determines the sub-base path, denoted as $B(t^{{}^{\prime }})$, which is an Euclidean path from $t^{\prime }$ to *t* that bypasses $H(t^{{}^{\prime }})$, H(t) and $C(\bar{t^{{}^{\prime }}},\bar{t})$ and satisfies either one of the following conditions:

- $B(t^{{}^{\prime }})$ is the shortest path from *t* to a vertex of C(t).
- $B(t^{{}^{\prime }})$ is $\left(\Gamma_{2}\times \left|L\left(t^{\prime },t\right)\right|\right)$-bounded path, where $L(t^{\prime },t)$ is the shortest path from $t^{\prime }$ to *t* and $\Gamma_{2}=Max\left\{1,\frac{L_{0}\left(1+\epsilon \right)}{\frac{\theta_{1}}{\sin\frac{(n-2)\pi }{2n}}\left(L_{0}+\frac{e_{C(t)}}{2}\right)+\frac{p_{C(t)}}{2}}\right\}$.

Let us denote $A(t^{{}^{\prime }})$ as the union of $H(t^{{}^{\prime }})$, H(t) and $C(\bar{t^{{}^{\prime }}},\bar{t})$, then $t^{\prime }$ constructs the so-called sub-Euclidean routing path (denoted as $R(t^{\prime },t)$) which is the image of $B(t^{{}^{\prime }})$ through the transformation $F(B\left(t^{{}^{\prime }}\right),A\left(t^{{}^{\prime }}\right),\alpha_{2})$, where $\alpha_{2}$ is a parameter defined by

$$\alpha_{2}=Max\left\{1,\frac{\left(1+\epsilon \right)L_{0}-\left|l_{t^{\prime }}\right|}{\left|B(t^{\prime })\right|}\right\}$$

where $l_{t^{{}^{\prime }}}$ is the routing path from *s* to $t^{\prime }$.

Finally, all the vertices of $R(t^{\prime },t)$ are inserted into the packets as virtual anchors. The packet then is greedily forwarded towards these virtual anchors gradually until reaching the destination *t*.
**Algorithm 2:** Euclidean routing path determination algorithm.**Input ***P*: *the set of base paths*, *s: source, d: destination*, ϵ*: stretch factor* **Output ***r: the Euclidean routing path*
B(s)← a base path probabilistically chosen from *P*;*s_core* ← the core polygon containing *s*;*s_hole* ← the hole whose core polygon is *s_core*;*d_core* ← the core polygon containing *d*;*d_hole* ← the hole whose core polygon is *d_core*;C← all core polygons excepting *s_core* and *d_core*;A←C;
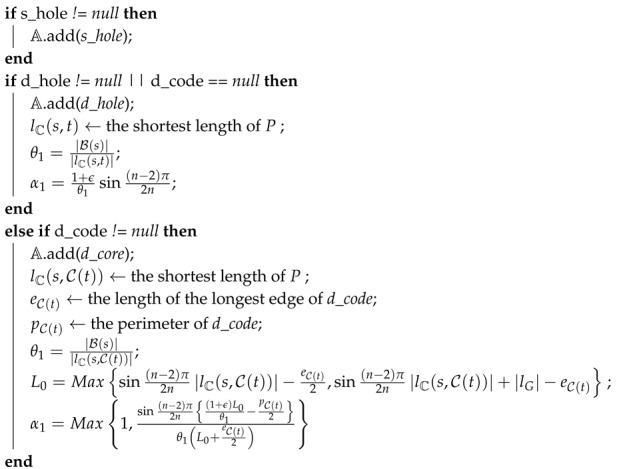
$r\leftarrow F(B\left(s\right),A,\alpha_{1})$;**return***r*

## 4. Theoretical Analysis

In this section, we analysis our proposed protocol theoretically. We denote our propose protocol as BSMH (i.e., load-Balanced and constant Stretch protocol for bypassing Multiple Holes). We first investigate the computational complexity of BSMH in Section 4.1 and then we present a preliminary and intuitive analysis about the impact of stretch factor, i.e., ϵ, in the performance of BSMH in Section 4.2.

### 4.1. Computational Complexity

In this section, we analysis the computational complexity of each phase of BSMH. Throughout this section, we denote *h* the total number of the holes in the network, *v* the largest number of the nodes belonging to the boundary of a hole, *n* the number of the vertices of a core polygon, $k_{n}$ the number of the core polygon candidates, *b* the maximum number of neighbors of a node.

#### 4.1.1. Complexity of the Hole Determination Algorithm

The hole determination algorithm consists of two steps: determining the stuck nodes (the nodes where local minimum phenomenon may happen and locating the hole boundary. The stuck node determination algorithm is conducted locally at every node. Specifically, every node first needs to sort its neighbor list by the clockwise order, and then it searches all its two adjacent neighbors to find stuck angles. As the number of neighbor is *b*, the computational complexity for sorting the neighbor list using famous sorting algorithms such as Heap sort, Quick sort, etc. is O(blogb); The computational complexity for searching all of the adjacent neighbor pairs is O(b). Consequently, the total computational complexity for determining the stuck nodes is O(blogb).

To locate the hole boundary, nodes on the hole boundary must forward HBA messages. To determine the next hop of an HBA message, the node performs RIGHT-HAND rule which investigates all neighbors of the current node to find the most-right neighbor. The computational complexity for determining the next hop by the RIGHT-HAND rule is O(b).

In consequence, the computational complexity of hole determination algorithm is O(blogb).

#### 4.1.2. Complexity of the Dissemination Algorithm

The node receiving an HCI packet may perform the following tasks:

1. Checking whether the node has already received an HCI packet.
2. Constructing the core polygon of the hole contained in the HCI packet.
3. Checking whether the node stays inside the vicinity region of the hole contained in the HCI packet.

To conduct the first task, the node needs to investigate all the core polygons and the holes stored in its memory. For each of them, the node identifies whether it is the same as the one contained in the HCI packet. The computational complexity for this task is O(h×v) (1).

In the second task, the node performs a loop over the vertices of the hole contained in the HCI packet. For each vertex, the node checks whether it is a core node (i.e., a node staying on the edges of the core polygon candidates). This job causes a computational complexity of $O(v\times n\times k_{n})$. Then, the node determines the vertices of all the core polygon candidates which are the intersection points of all lines going through the core nodes and making specific angles with the *x*-axis. This job requires a complexity of $O(n\times k_{n})$. Finally, the node computes the area of each core polygon candidate and chooses the core polygon candidate with the smallest area to be the core polygon. The computational complexity of this job is $O(n\times k_{n})$. In consequence, the total complexity of the second task is $O(v\times n\times k_{n})$ (2).

In the third task, the node just needs to compute the distances to the core polygon stored in the HCI packet and compare them with the threshold value. Thus, the computational complexity of this task is O(n) (3).

From (1), (2) and (3), it is deduced that the computational complexity of the dissemination algorithm is $O(v\times \left(h+nk_{n}\right))$.

#### 4.1.3. Complexity of the Data Forwarding Algorithm

To forward a data packet, the node may need to perform the following tasks:

1. Determining the base path candidate set (i.e., in the case that the node is the source node).
2. Determining the Euclidean routing path (i.e., in the case that the node is the source node).
3. Determining the next hop by using the greedy forwarding algorithm.

To determine the base path candidates, the node needs to identify the hole containing the source and the destination. This job requires the computational complexity of O(h×n). Then, the node constructs the visibility graph whose vertices are the vertices of the core polygons that do not containing the source and the destination, and the vertices of the holes containing the source and the destination, and whose edges do not intersect the interior of the core polygons and holes. As the number of the vertices of the visibility graph does not exceed 2v+(h−2)n, the computational complexity for constructing such a visibility graph does not exceed $O\left({\left(2v+(h-2)n\right)}^{3}\right)$. Based on the visibility graph, the node determines all the base path candidates by using breath-first search whose computational complexity is $O\left(2v+\left(h-2\right)n+{\left(2v+(h-2)n\right)}^{2}\right)$. Therefore, the computational complexity of the first task (i.e., finding the base path candidates) is $O\left(h\times n+{\left(2v+(h-2)n\right)}^{3}+2v+\left(h-2\right)n+{\left(2v+(h-2)n\right)}^{2}\right)$ which is equivalent to $O\left({\left(2v+(h-2)n\right)}^{3}\right)$ (4).

Let *c* be the number of the base path candidates, then the complexity for choosing a base path from the candidate list is O(c). To scale up the base path using the homothetic transformation, the node needs to determine the transformation centers, scale factors and the coordinates of the images. The computational complexity for these jobs is O(m), where *m* is the number of vertices of the base path. As m<2v+(h−2)n, the computational time complexity for the second task does not exceed O(c+2v+(h−2)n) (5).

Having determined the Euclidean path, the next hop is determined by using greedy algorithm whose computational complexity is only O(b), where *b* is the number of 1-hop neighbors (6).

From (4), (5), and (6), the time complexity of data forwarding algorithm is $O\left({\left(2v+(h-2)n\right)}^{3}+c+b\right)$.

### 4.2. Impact of the Stretch Factor

#### 4.2.1. Routing Path Stretch

In BSMH, packets are forwarded along the Euclidean routing path which is the image of the base path through the homothetic transformations. As described in Section 3.3.2, the scale factor of the homothetic transformation is defined by:

$$\alpha_{1}=\left\{\begin{array}{c} \frac{1+\epsilon }{\theta_{1}}\sin\frac{(n-2)\pi }{2n},\;\mathrm{if}\;s\;\mathrm{is}\;a\;t-\mathrm{aware}\;\mathrm{node}\\ Max\left\{1,\frac{\sin\frac{(n-2)\pi }{2n}\left\{\frac{\left(1+\epsilon \right)L_{0}}{\theta_{2}}-\frac{p_{C(t)}}{2}\right\}}{\theta_{1}\left(L_{0}+\frac{e_{C(t)}}{2}\right)}\right\},\;\mathrm{otherwise} \end{array}\right.$$

Obviously, $\alpha_{1}$ is proportional with ϵ. It means that the greater the ϵ, the longer the Euclidean routing path. Therefore, when ϵ increases, the routing path stretch will be increased too.

#### 4.2.2. Load Balance

The relationship between ϵ and the network’s load balance is not straightforward. In the following, we present a preliminary and intuitive theoretical analysis of this relationship.

**Figure 3:**
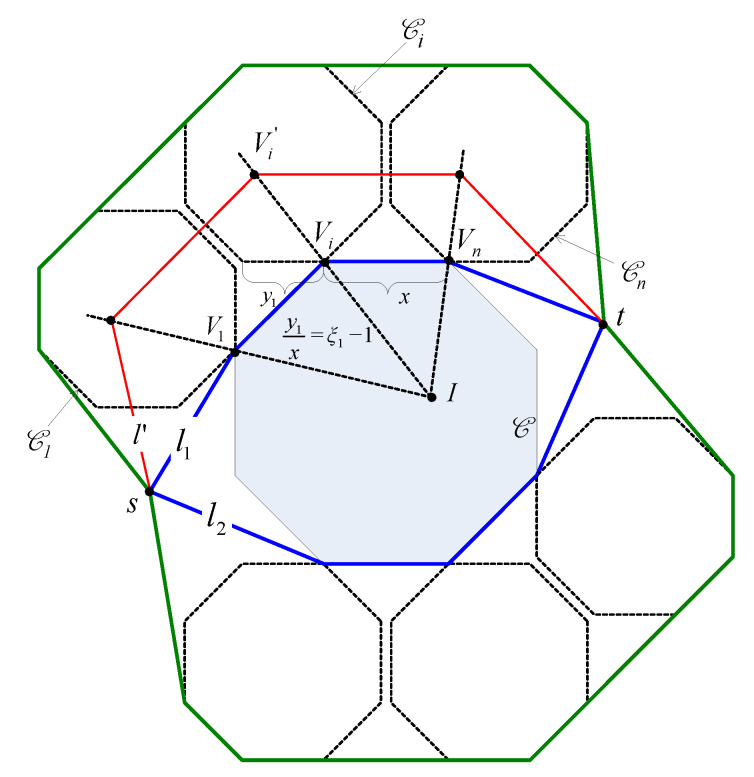
The trajectory of Euclidean routing paths. The base paths $l_{1}$ and $l_{2}$ are colored blue, the Euclidean path $l^{\prime }$ is colored red [38].

Let us consider a simple network topology consisting of only one hole and one source-destination pair as shown in Figure 3. Let us denote by C the hole, by *s* the source node and by *t* the destination node. Denote by $l_{1}$ and $l_{2}$ the two Euclidean paths that bypass C and that have all the vertices be the vertices of C as shown in Figure 3. Suppose that $l_{1}$ is shorter than $l_{2}$. Then, if ϵ is sufficiently large, the base path candidates are both $l_{1}$ and $l_{2}$. Otherwise, the base path candidates consist of only $l_{1}$. Suppose $l^{\prime }$ is a Euclidean path. Without loss of generality, we assume that $l^{\prime }$ is the image of $l_{1}$ through a homothetic transformation with the center *I* and the scale factor ξ.

**Figure 4:**
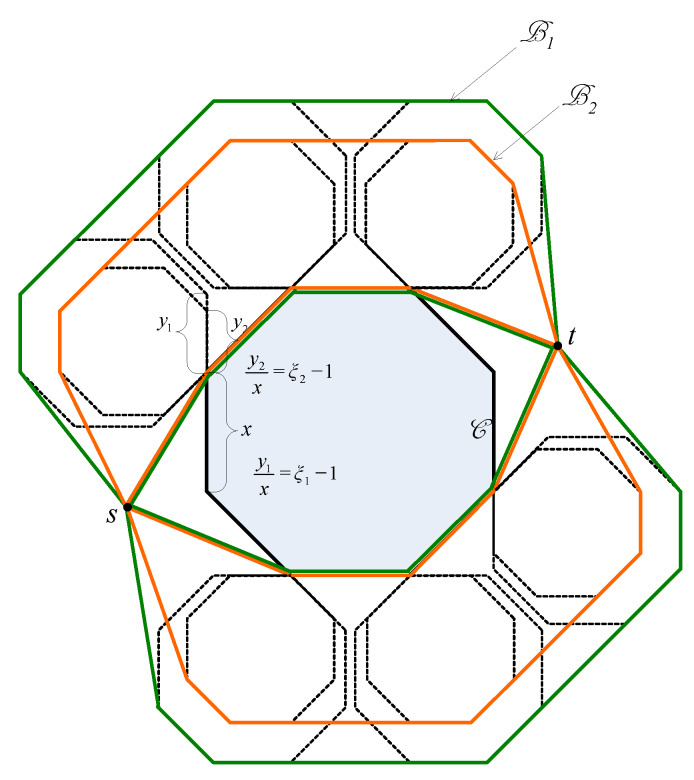
The trajectories of Euclidean routing paths with different values of ϵ. The trajectory of Euclidean routing paths when $\epsilon =\epsilon_{1}$, is bounded by the green line. The trajectory of Euclidean routing paths when $\epsilon =\epsilon_{2}$, is bounded by the orange line [38].

Please note that ξ is defined by Formula (1), and it can be written as:

(2) $$\xi =\gamma_{1}\epsilon +\gamma_{2}$$

where $\gamma_{1}$ and $\gamma_{2}$ are constants that the same for all packets from *s* to *t*. Suppose $V_{1},\ldots ,V_{n}$ are the vertices of C that are vertices of $l_{1}$. Let $V_{i}^{{}^{\prime }}$ be the image of $V_{i}$ through the homothetic transformation (∀i=1,…,n). Then, obviously $V_{i}^{{}^{\prime }}$ is the image of *I* through the homothetic center with the center $V_{i}$ and the scale factor ξ−1. As *I* locates inside C, $V_{i}^{{}^{\prime }}$ must stay inside the image of C through the homothetic transformation with the center $V_{i}$ and the scale factor ξ−1 (denoted as $C_{i}$) (see Figure 3). Let B be the union of all $C_{i}$ (∀i=1,…,n). Please note that when ϵ is sufficiently small such that B stays inside the network, then B is the trajectory of all routing paths from *s* to *t*. Accordingly, the trajectory of sensor nodes that participate in forwarding packets are polygons that are limited by the boundaries of $C_{i}$ and the base paths. In Figure 3, this trajectory are polygons whose boundaries are the green lines and the blue lines. Let $\epsilon_{1}$ and $\epsilon_{2}$ are two positive number where $\epsilon_{1}>\epsilon_{2}$. Let $B_{1}$ and $B_{2}$ be the trajectories of sensor nodes participating in forwarding packets when $\epsilon =\epsilon_{1}$, and $\epsilon =\epsilon_{2}$, respectively. Figure 4 illustrates $B_{1}$ and $B_{2}$, in which the boundary of $B_{1}$ is colored orange and the boundary of $B_{1}$ is colored green. In the following we will prove the following statement: *“If $B_{1}$ and $B_{2}$ stay inside the network, then the probability for a node to forward a packet in the case $\epsilon =\epsilon_{1}$ does not exceed that in the case $\epsilon =\epsilon_{2}$”*.

Denote $\xi_{1}$ and $\xi_{2}$ the scale factors in the homothetic transformations in the cases $\epsilon =\epsilon_{1}$ and $\epsilon =\epsilon_{2}$, respectively. According to Formula (2), $\xi_{1}$ and $\xi_{2}$ is proportional to $\epsilon_{1}$ and $\epsilon_{2}$, respectively. Let $R_{1}$ be a Euclidean routing path obtained in the case $\epsilon =\epsilon_{1}$. Then, obviously, there must exist such a Euclidean routing path $R_{2}$ obtained in the case $\epsilon =\epsilon_{2}$ that $R_{1}$ is an image of $R_{2}$ through a homothetic transformation with the scale factor $\frac{\xi_{1}}{\xi_{2}}$ (because they are image of a base path through homothetic transformations with the scale factors of $\xi_{1}$ and $\xi_{2}$, respectively). It is trivial that the length of $R_{1}$ does not exceed $\frac{\xi_{1}}{\xi_{2}}$ times that of $R_{2}$. Consequently, let $T_{1}$ and $T_{2}$ be the total hopcounts of all packets from *s* to *t* in the cases $\epsilon =\epsilon_{1}$ and $\epsilon =\epsilon_{2}$, respectively. Then, we can deduce that:

(3) $$L_{1}\leq \frac{\xi_{1}}{\xi_{2}}L_{2}$$

**Figure 5:**
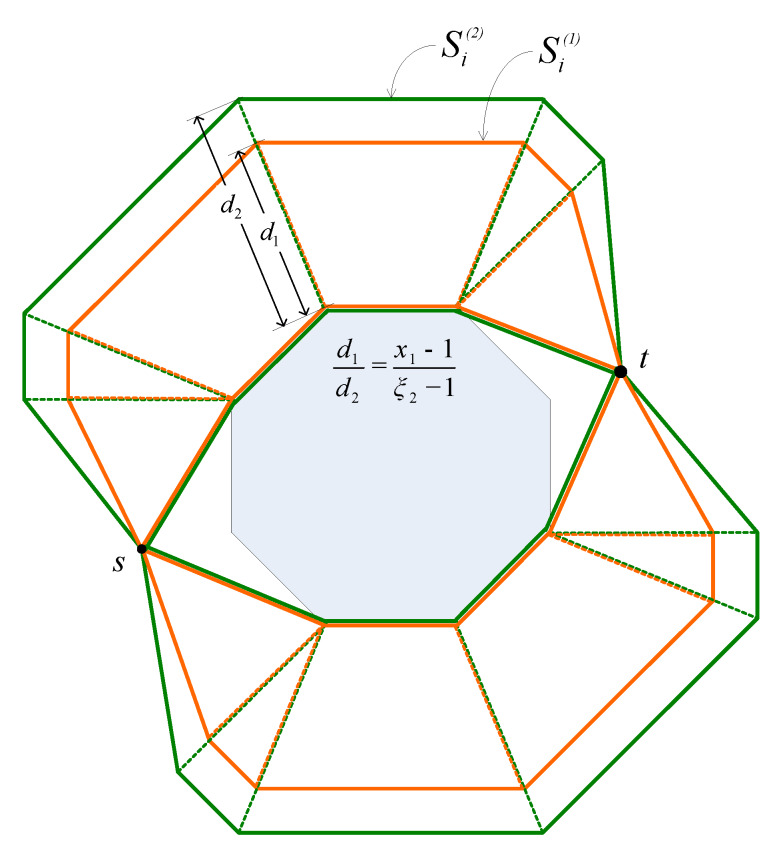
Comparison of the areas of $B_{1}$ and $B_{2}$. In order to compare $B_{1}$ and $B_{2}$, we divide them into sub-regions by dotted lines [38].

Moreover, let us divide $B_{1}$ and $B_{2}$ into sub-regions by the segments connecting their vertices as shown in Figure 5. Denote the i-th sub-region of $B_{1}$ as $S_{i}^{\left(1\right)}$ and the corresponding sub-region of $B_{2}$ as $S_{i}^{\left(2\right)}$ (Figure 5). Obviously, the area of $S_{i}^{\left(1\right)}$ is $\frac{\xi_{1}-1}{\xi_{2}-2}$ times that of the area of $S_{i}^{\left(2\right)}$. Let $n_{1}$ and $n_{2}$ be the number of nodes participating in forwarding packets in the case $\epsilon =\epsilon_{1}$ and $\epsilon =\epsilon_{2}$, respectively. If we assume that the nodes are evenly distributed in the network, then $n_{1}$ and $n_{2}$ are proportional to the areas of $B_{1}$ and $B_{2}$, respectively. Accordingly, we have the following:

(4) $$n_{1}=\frac{\xi_{1}-1}{\xi_{2}-1}n_{2}$$

Denote by $p_{1}$ and $p_{2}$ the probabilities for a node to forward a packet in the cases $\epsilon =\epsilon_{1}$ and $\epsilon =\epsilon_{2}$, respectively. Then, we have:

(5) $$\begin{array}{ccc} p_{1} & = & \frac{L_{1}}{n_{1}} \end{array}$$

(6) $$\begin{array}{ccc} p_{1} & = & \frac{L_{2}}{n_{2}} \end{array}$$

From Equations (3)–(5), it can be deduced that $p_{1}\leq p_{2}$. It means that when ϵ is sufficiently small such that the trajectory of all routing paths from *s* to *t* stays inside the network, then increasing ϵ tends to improve the load balance.

**Figure 6:**
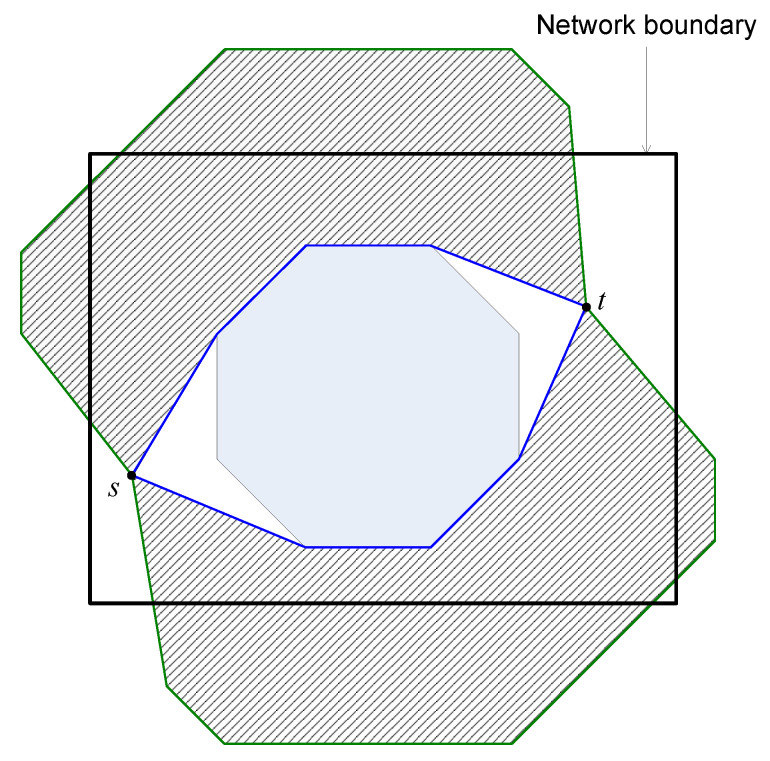
When ϵ is too large, the trajectory of the Euclidean routing paths may exceed the network boundary [38].

However, when ϵ is too large, the trajectory of all routing paths from *s* to *t* may exceed the network boundary as shown in Figure 6. In this case, the area of the trajectory becomes almost stable when increasing ϵ. It means that increasing ϵ cannot increase the number of sensors participating in the routing process. In the meanwhile, the routing path stretch still gradually increases when increasing ϵ. Consequently, increasing ϵ tends to decrease the load balance.

## 5. Performance Evaluation

### 5.1. Performance Metrics

We evaluate and compare the performance of BSMH with three existing protocols, namely GPSR [23] and LVGR [34] and EDGR [45]. GPSR is a typical perimeter routing protocol, which uses greedy forwarding as the default mode. When a packet encounters the local minimum phenomenon, it switches from greedy to perimeter mode whereby the packet is forwarded along the hole boundary. In LVGR, the packet is forwarded along a hole-bypassing Euclidean path from the source to the destination. This path is determined by using a visibility graph whose vertices are the vertices of the convex hulls of the holes. In EDGR, the source nodes randomly choose an anchor lists from two candidates. The packet then is forwarded towards the chosen anchor lists, gradually. Moreover, the next hop is heuristically chosen based on not only location information but also the residual energy. We focus on three design factors: routing path-length minimization, control overhead minimization and load balance maximization. In this section, we investigate how does our proposed protocol satisfy these design factors by using common evaluation metrics which have been used in many other related works [50,51,52,53,54].

1. **Metrics regarding routing path-length minimization**
  - *Average routing path stretch:* The routing path stretch of a routing path is defined by the ratio of its hop count to that of the theoretical shortest path. The average routing path stretch of a routing protocol is the average value of routing path stretches of all the routing paths determined by the protocol.
  - *Average delay:* We evaluate the average end-to-end delay of all data packets that successfully arrive at the destinations.
2. **Metrics regarding control overhead minimization**
  - *Total amount of control packets:* Control packets are defined as packets that are not data packets. In BSMH and LVGR, control packets consist of packets for exchanging node information (known as HELLO packets), packets for determining hole boundaries (known as HBA packets), and packets for broadcasting hole information (known as HCI packets). In GPSR, the control packet is only HELLO packet. In EDGR, control packets include beacons that periodically broadcast node information (i.e., energy, location, ...), and burst packets that determine the anchors. We measure the total amount (in bytes) of all the control packets which has been transmitted from when the simulation starts till the first node dies.
3. **Metrics regarding load balance maximization**
  - *Maximum packet forwarding ratio:* This indicates the maximum ratio of the number of packets forwarded by a node to the total number of packets sent. Specifically, let $p_{i}$ be the number of packets forwarded by the i-th node, and *p* be the total number of packet sent by all source nodes, then the maximum forwarding ratio is defined by $Max\left\{\frac{p_{i}}{p}\right\}$. In general, the smaller maximum packet forwarding ratio reflects the better load balance.
  - *Network lifetime:* As described in Section 1, in large-scale wireless sensor networks, the death of even only one node may affect the operation of the whole network. Accordingly, the network lifetime should be defined as the time period until the first node dies. In our experiments, all protocols spend the first 400s for the setup phase, and the first data packet is sent after that. Thus, we define the network lifetime as the time period from the first data packet was sent until the first node dies.

Besides the above metrics, the following metrics are also used because they are the common metrics which have been used frequently in evaluating routing protocols in WSNs.

4. **Other metrics**
  - *Delivery ratio:* This is defined by the ratio of the number of data packets successfully arriving at the destinations to the total number of data packets sent by the sources.
  - *Energy consumption per packet:* This is the ratio of the total energy consumption of all the nodes to the total number of packets successfully delivered.

### 5.2. Simulation Settings

We use various network topologies that are generated based on the real map obtained from the Google Earth as follows. First, we extract from the maps around the Amazon ten regions which contain obstacles, and embed each them into a 1000 m × 1000 m network area. Then, for each network area, we randomly scatter about 4000 nodes by dividing the network into 63×63 square grid and in each square, we put one sensor in a random position. Finally, we remove all sensor nodes that stay inside the obstacles. Please note that we have also performed the simulation with various settings concerning the network size, the number of sensors, etc., We observed that the results obtained in all the scenarios have similar trends. Thus, in this paper, we present the results of a particular case.

**Figure 7:**
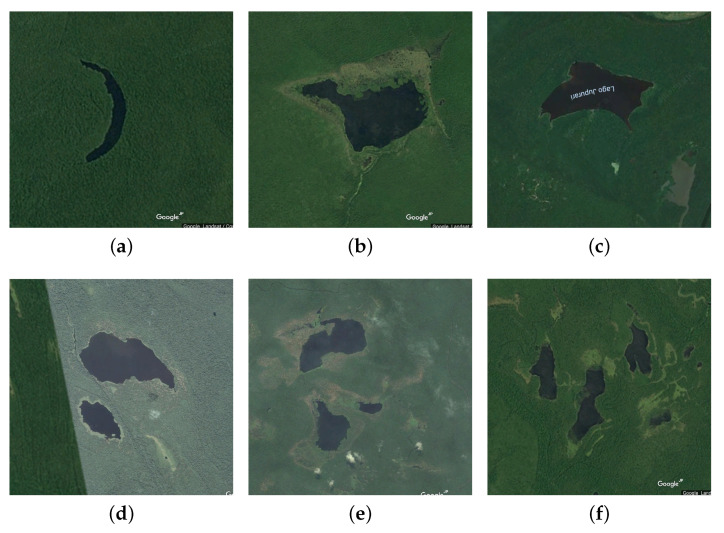
Real maps obtained from the Google Earth. The black regions represent the lakes [38]. (**a**) Region 1. (**b**) Region 2. (**c**) Region 3. (**d**) Region 4. (**e**) Region 5. (**f**) Region 6.

**Figure 8:**
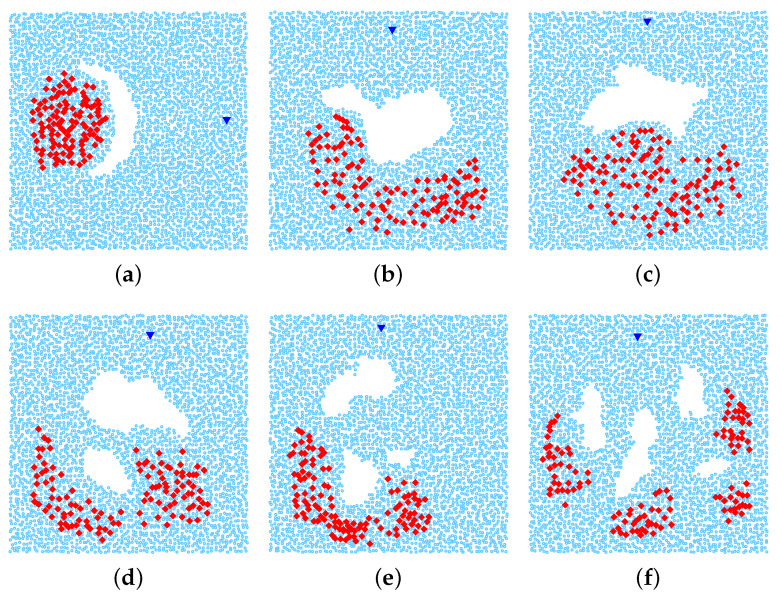
Network topologies for 1-n communication. (**a**) Topology 1. (**b**) Topology 2. (**c**) Topology 3. (**d**) Topology 4. (**e**) Topology 5. (**f**) Topology 6.

**Figure 9:**
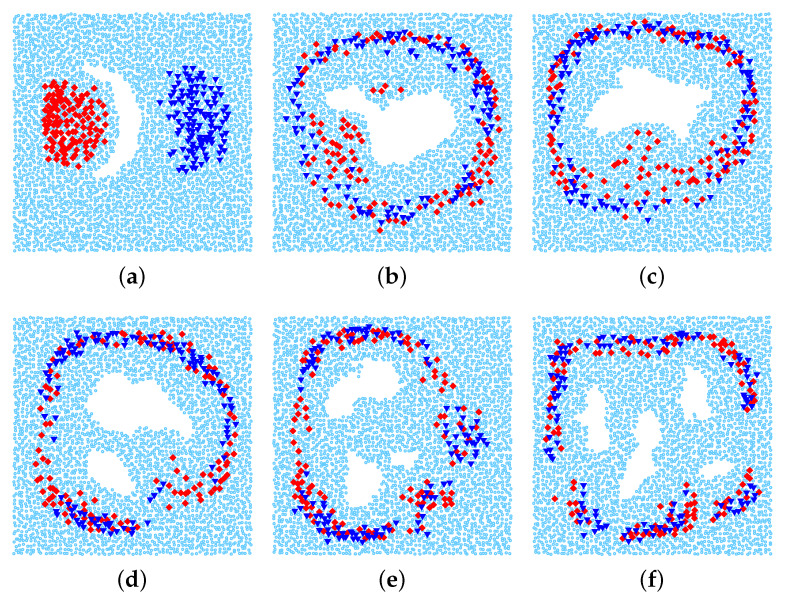
Network topologies for n-n communication [38]. The blue circles represent the sensors. The red diamonds represent the sources and the blue triangle represents the destination. (**a**) Topology 1. (**b**) Topology 2. (**c**) Topology 3. (**d**) Topology 4. (**e**) Topology 5. (**f**) Topology 6.

Figure 7 shows the real images obtained from the Google Earth. From these images, we create six network topologies for 1-n communication (multiple sources vs one destination) (Figure 8) and other sixes topologies for n-n communication (multiple sources vs multiple destinations) (Figure 9). There are total 120 source-destination pairs which are randomly chosen to intersect with the holes.

The simulated time is 2000 s. The first 400 s is for network setup and the remaining 1600 s is for data forwarding. In the network setup phase, the nodes broadcast HELLO packets which contain their own location information. In LVGR and BSMH, the network setup phase is also for locating and disseminating information of the holes and the forbidden areas. The plotted values are the average of 10 simulation runs along with 95% confidence interval. The experiments are conducted using the NS-2 simulator and on a computer with an Intel Core i5-4570 3.2 GHz × 4 CPU and 8 GB of RAM, and running Ubuntu 14.04 64-bit. The MAC protocol, interface queue model, radio model, antenna type, queue length, transmission range are set to the default values of NS2. To study the energy consumption of the protocols, we used the energy model suggested by Shnayder et al. [55]. Specifically, the power supply is set to 3 V, the currents regrading idle state, receiving state and transmitting state are set to 3.2 mA, 15 mA and 21.5 mA, respectively. Accordingly, the per second energy consumption with respect to idle state, receiving state and transmitting state are 9.6 mW, 45 mW and 88.5 mW, respectively. The initial energy of all node is set to 30 J to ensure that the network lifetimes achieved by all protocols are smaller than the simulated time. As the maximum size of a packet following 802.15.4 standard is 127 bytes, if the packet size exceeds 127 bytes, there will be multiple packets sent. Therefore, the data packet size should be set to less than 127 bytes. We have conducted the experiments with the data packets size of 50 bytes and 100 bytes and found that the trend of the results does not depend on the data packet size. Therefore, in the following, we set the size of all data packets to 50 bytes. The fragmentation threshold of the control packets is set to the default value in NS2, i.e., 1000 bytes. When the size of a control packet exceeds the fragmentation threshold, it will be fragmented into multiple packets. Table 1 summarizes the parameters used for the sensor nodes.

### 5.3. Impact of the Stretch Factor ϵ

In this section, we study the impact of the stretch factor, ϵ, in the performance of BSMH. To do so, we vary the value of ϵ from 0.3 to 4.5 and observe its impact on the routing path stretch and load balance achieved by BSMH. Although the experiments are conducted on all topologies shown in Figure 8 and Figure 9, to facilitate the readability, in what follows, we show only the results on one topology with single hole, i.e., Topology 2 (Figure 8b).

**Figure 10:**
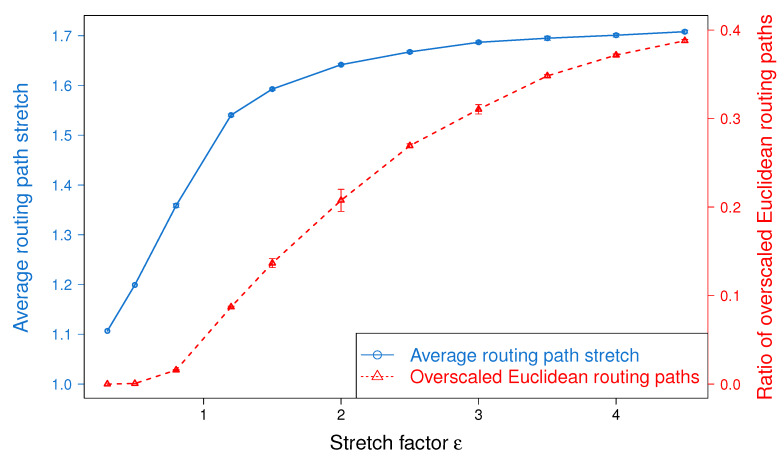
Impact of ϵ on routing path stretch. The blue lines represent the average stretch of all routing paths, the red lines represent the percentage of Euclidean routing paths that exceed the network boundary.

Figure 10 depicts the impact of ϵ on the routing path stretch. As shown, the average routing path stretch increases gradually when ϵ increases. This phenomenon can be explained as follows. In BSMH, packets are forwarded along the Euclidean paths that are the image of the base paths through homothetic transformations. The scale factor of the homothetic transformations is proportional to ϵ. Therefore, the increase of ϵ leads to the enlargement of the Euclidean paths, and increase the routing path stretch, consequently.

**Figure 11:**
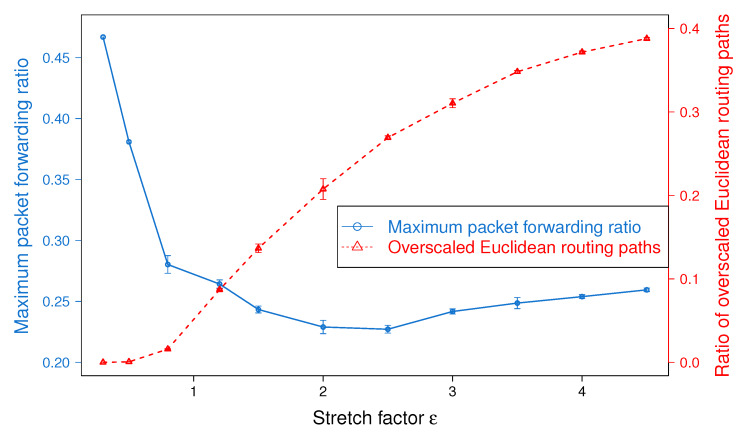
Impact of ϵ on load balance. The blue lines represent the maximum packet forwarding ratio of sensor nodes, the red lines represent the percentage of Euclidean routing paths that exceed the network boundary.

The impact of ϵ on the maximum packet forwarding ratio is shown in Figure 11. As shown, when ϵ increases from 0.3 to 0.8, almost all the Euclidean routing paths stay inside the network. Therefore, increasing ϵ helps to enlarge the number of sensors participating in forwarding packets, thereby reduce the maximum packet forwarding ratio. When ϵ varies from 0.8 to 2.0, several Euclidean routing paths may exceed the network boundary, thus the increase of ϵ almost cannot help to improve the load balance (i.e., the maximum forwarding ratio is almost stable when ϵ varies in this range). The greater ϵ, the more Euclidean routing paths exceeding the network boundary. Specifically, when ϵ reaches 2.5, more than 25% of Euclidean routing paths staying outside of the network boundary. Hence, increasing ϵ cannot enlarge the number of sensors participating in forwarding packets. In the meanwhile, the increase of ϵ results in the increase of routing path length (see Figure 10), thus imposes more traffic load on the sensor nodes. Consequently, when ϵ≥2.5, the increase of ϵ worsen the load balance.

**Figure 12:**
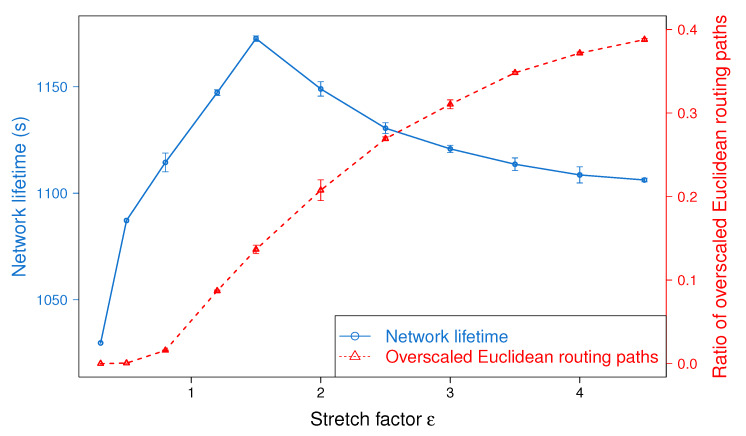
Impact of ϵ on the network lifetime. The blue lines represent the network lifetime, the red lines represent the percentage of Euclidean routing paths that exceed the network boundary.

Figure 12 illustrates the impact of ϵ on the network lifetime. The trend of the network lifetime is similar to that of the load balance, but they are not exactly the same. As shown, the network lifetime gets the peak at ϵ=1.5. Before the peak, the increase of ϵ extends the network lifetime significantly, but beyond the peak, the network lifetime decreases fast when increasing ϵ. This phenomenon can be explained as follows. When ϵ≤1.5, the increase of ϵ improve the load balance significantly (this can be seen through the big slope in Figure 11), thus the network lifetime is extended. When ϵ varies from 1.5 to 2.5, the load balance stays almost the same, but the average routing path stretch of all routing paths increases (as shown in Figure 10). Please note that besides the energy consumed for sending packets, nodes must spend energy for receiving packets from its neighbors. The increase of average routing path stretch results in the enlargement of the total traffic in the network, which may increase the energy consumed for packet receiving. In consequence, the network lifetime decreases when ϵ varies from 1.5 to 2.5. Beyond 2.5, increasing of ϵ not only increases the routing path stretch, but also worsens the load balance, thus the network lifetime is shortened.

Finally, it can be seen that the experimental results show the consistency with the theoretical analysis described in Section 4.2.

### 5.4. Comparison of BSMH and Other Benchmarks

In the following sections, we will compare the performance of our protocol with those of three existing protocols, namely GPSR [23] and LVGR [34] and EDGR [45]. GPSR uses greedy forwarding as the default mode. When a packet encounters the local minimum phenomenon, it switches from greedy to perimeter mode whereby the packet is forwarded along the hole boundary. In LVGR, the packet is forwarded along a hole-bypassing Euclidean path from the source to the destination. This path is determined by using a visibility graph whose vertices are the vertices of the convex hulls of the holes. In EDGR, the source nodes randomly choose an anchor lists from two candidates. The packet then is forwarded towards the chosen anchor lists, gradually. Moreover, the next hop is heuristically chosen based on not only location information but also the residual energy.

The stretch factor (i.e., ϵ) of BSMH is set to 0.3, 0.5 and 1.2, the number of vertices of the core polygons is set to 8.

#### 5.4.1. Average Routing Path Stretch

**Figure 13:**
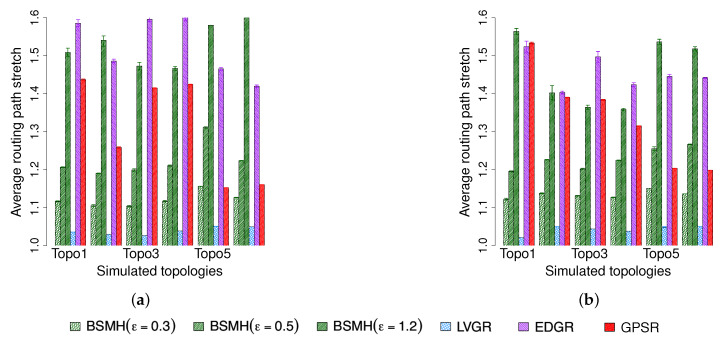
The average routing path stretch of successfully delivered packets [38]. (**a**) 1-n communication. (**b**) n-n communication.

The comparison in terms of the average routing path stretch is shown in Figure 13. As shown, for all network topologies, LVGR attains the best performance and followed by BSMH with ϵ=0.3. However, as will be shown in Section 5.4.6 and Section 5.4.7, the shortest routing path stretch of LVGR has to compensate by a poor performance regarding the load balance and the network lifetime. In general, BSMH with small values of ϵ (i.e., ϵ=0.3;0.5) attains smaller routing path stretch than EDGR and GPSR. However, the increase of ϵ will increases the routing path stretch, and when ϵ=1.2, the routing path stretch of BSMH is higher than that of EDGR and GPSR in 8/12 cases. More specifically, we have the following detail observations. Regarding to the 1-n communication, the average routing path stretch attained by BSMH with ϵ=0.3 is always less than 79% that of EDGR in all topologies, and 97% that of GPSR in 5/6 topologies (excepting Topology 5). When ϵ reaches 0.5, BSMH results in the average routing path stretch which is smaller than 89% that of EDGR in all cases, and smaller than 97% that of GPSR in 4/6 cases (in the other cases, the average routing path stretch caused by BSMH and GPSR are almost similar). Regarding the n-n communication, the average routing path stretch attained by BSMH with ϵ=0.3 is less than 81% that of EDGR and 96% that of GPSR in all topologies. In the best cases, the BSMH gains the average routing path stretch begin smaller than 75% that of EDGR and 72% that of GPSR. When ϵ=0.5, the average routing path stretch caused by BSMH is always less than 89% that of EDGR, and less than 93% that of GPSR in 4/6 cases (in the other cases, the average routing path stretch caused by BSMH and GPSR are almost similar).

#### 5.4.2. Average End-to-End Delay

**Figure 14:**
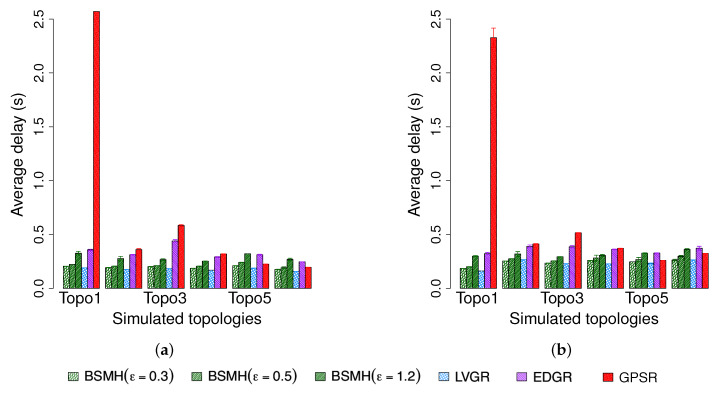
Average end-to-end delay of successfully delivered packets. (**a**) 1-n communication. (**b**) n-n communication.

Figure 14 depicts the average end-to-end delay of all successfully delivered packets. Since LVGR attains the best performance in terms of the average routing path stretch, it also results in the smallest average end-to-end delay. Following LVGR, BSMH with small values of ϵ (i.e., ϵ=0.3 and 0.5) gains the second-best performance. Moreover, the performance gap between LVGR and BSMH is insignificant when ϵ is small. For examples, when ϵ=0.3, the average end-to-end delay resulted by BSMH is smaller than 1.1 times that caused by LVGR in both 1-n and n-n communication. Similar to the routing path stretch, the end-to-end delay caused by BSMH increases with the increase of ϵ. When ϵ reaches 1.2, BSMH may cause an end-to-end delay higher than 1.7 time that of LVGR in the worst cases (i.e., Topo 3, 4 in 1-n communication). In comparison with EDGR, it can be seen that the end-to-end delay caused by BSMH with ϵ=0.3 and 0.5 are smaller than that of EDGR in all topologies. Specifically, BSMH with ϵ=0.3 results in an average routing path stretch smaller than 71% and 75% regrading 1-n and n-n communications, respectively. When ϵ increases to 0.5, the ratio between the routing path stretch of BSMH to that of EDGR is smaller than 0.78 and 0.81 with respect to 1-n and n-n communications, respectively. The performance of GPSR strongly depends on the network topology. Specifically, its end-to-end delay is extremely higher than those of the other protocols in some cases such as Topologies 1 and 3 despite the fact that its average routing path stretch is not much higher than that of the other protocols. We observe that the end-to-end delay is affected not only by the transmission time between the hops, but also is contributed by the processing time in the hops. While the former one closely related to the routing path routing path stretch, the latter one has a strong relationship with the load balance. Hence, there are two main reasons causing the worse performance of GPSR in terms of end-to-end delay. The first reason is due to the long route path (as described in Section 5.4.1), and the second reason is due to the traffic concentration around the hole boundaries (which has been shown in Section 5.4.6).

#### 5.4.3. Control Overhead

**Figure 15:**
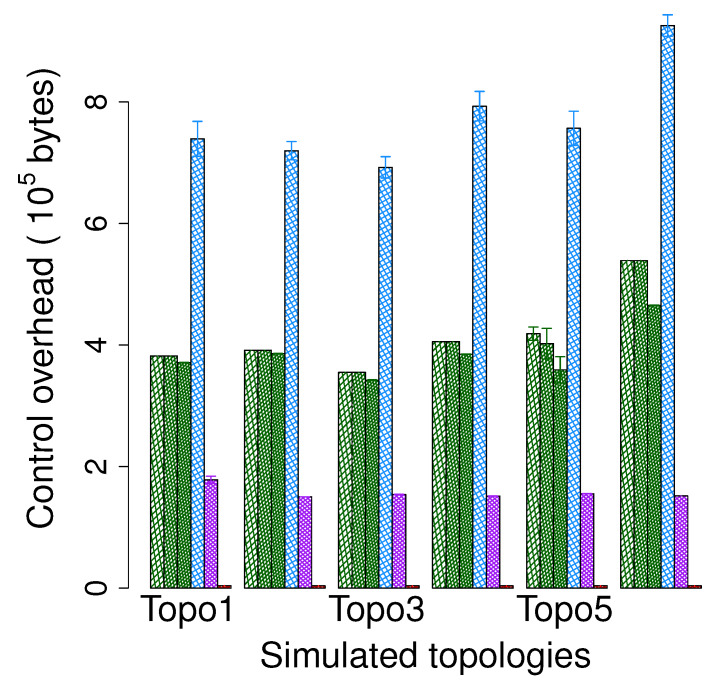
Total amount of control packets [38].

The total amount of control packets required by the protocols is presented in Figure 15. As the control overhead does not depend on the communication type, we plot the results regarding 1-n communication.

As expected, GPSR achieves the best performance and its overhead is extremely smaller than those of the others. Although EDGR requires the sending of the beacons periodically, these beacons are quite lightweight, therefore the overhead caused by EDGR is still smaller than those of BSMH and LVGR. Both BSMH and LVGR need to determine the hole boundaries and disseminate hole information. Moreover, since the control packets used in these two phases are quite heavy, BSMH and LVGR result in the worst performance. However, note that the hole boundary determination and hole information dissemination phases of BSMH and LVGR are conducted only one time while the beaconing of EDGR is conducted periodically, the control overhead of EDGR may higher than those of BSMH and LVGR when increasing the simulation time. By using core polygon instead of the exact hole, BSMH reduces overhead significantly compared with LVGR. Specifically, the overhead caused by BSMH is always smaller than 53% that caused LVGR.

Another observation is that the overhead of BSMH slightly decreases when increasing the ϵ. This is due to the fact that the increase of ϵ will shrink the region for disseminating hole information.

#### 5.4.4. Energy Consumption per Packet

**Figure 16:**
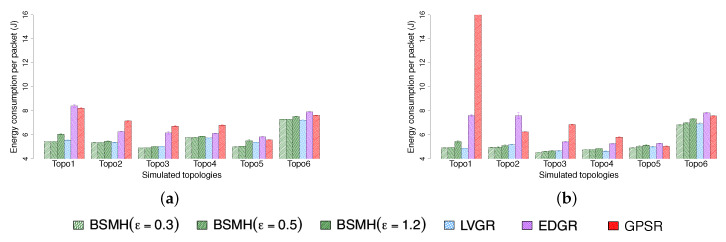
Average energy consumption per packet. (**a**) 1-n communication. (**b**) n-n communication.

The average energy consumed to delivery one packet is shown in Figure 16. In general, BSMH and LVGR outperform EDGR and GPSR. Moreover, the performance of BSMH and LVGR are almost similar. GPSR shows the worst performance and its energy consumption per packet is much higher than those caused by other protocols in most the cases. EDGR results in a poor performance regarding Topologies 1, 2, 3, 4. This is due to its high delivery ratios and large routing path stretch (see Section 5.4.1 and Section 5.4.5). In the other topologies, EDGR attains a similar performance with BSMH and LVGR. This phenomenon can be explained as followed: As BSMH and LVGR use shorter routing paths (reflected by better performance in terms of routing path stretch as shown in Section 5.4.1), they may consume less energy than EDGR regarding data transmission. However, as will be shown in Section 5.4.3, the control overhead caused by EDGR is much smaller than those of BSMH and LVGR, thus the energy consumed by transmitting the control packets of EDGR is smaller than those of BSMH and LVGR.

#### 5.4.5. Packet Delivery Ratio

**Figure 17:**
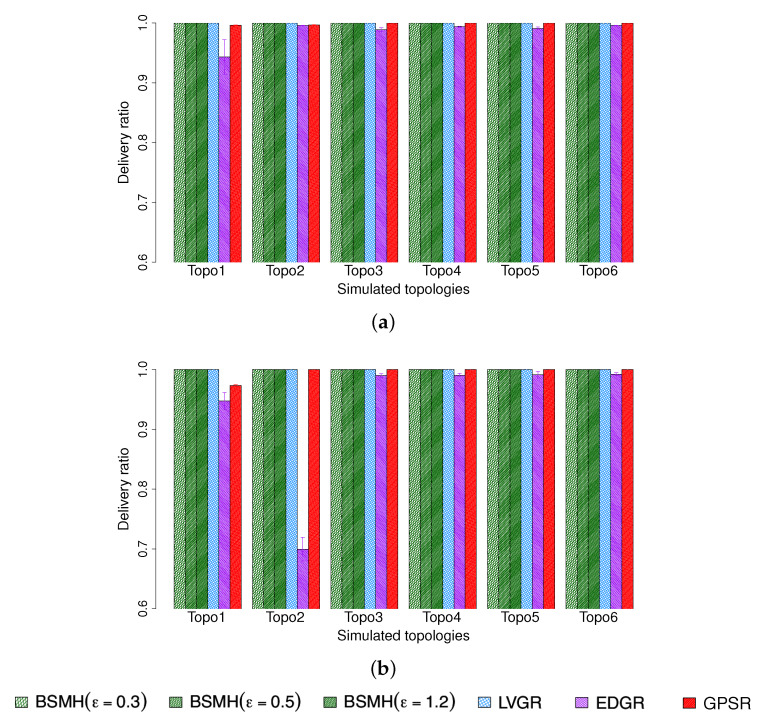
The ratio of the number of packets successfully delivered to the total number of packets sent. (**a**) 1-n communication. (**b**) n-n communication.

Figure 17 depicts the delivery ratio of the protocols. As shown, BSMH and LVGR achieve the best performance with the delivery ratio approximately equals to 1 in both 1-n and n-n communication types. The reason is because BSMH and LVGR forward all packets along hole-bypassing paths. As hole-bypassing paths do not intersect the holes’ interior, the local minimum problem is solved thoroughly, and all packets are delivered to the destinations. GPSR achieves a good performance in topologies where the sources stay outside of concave areas of the holes, e.g., Topologies 4, 5, 6. When the sources stay inside of concave areas of the holes, e.g., Topologies 1, 2, GPSR suffer from local minimum problem and some packets are dropped. In all the cases, the delivery ratio of GPSR is higher than 97%.

EDGR attains a high delivery ratio in Topologies 3, 4, 5, 6, but its performance degrades severely in Topologies 1, 2. In the worst case, i.e., Topo 2 in n-n communication, EDGR can delivery only less than 70% of packets to the destinations. We observe that the main reason for packet dropping of EDGR is because the burst packets (which are used to determine the anchor list) could not come back to the source before sending data packets.

#### 5.4.6. Maximum Packet forwarding Ratio

**Figure 18:**
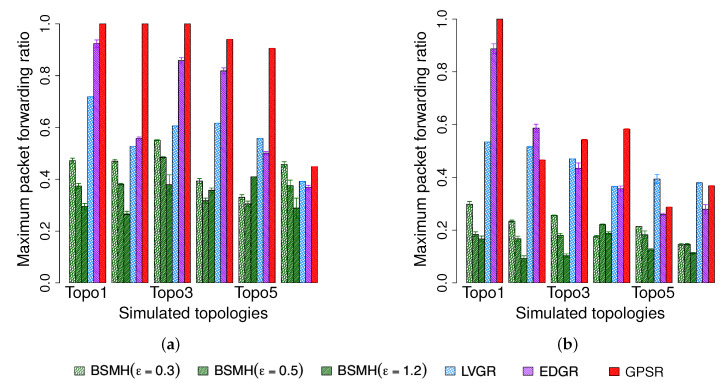
The maximum percentage of packets forwarded by a sensor node [38]. (**a**) 1-n communication. (**b**) n-n communication.

The maximum packet ratios caused by the protocols are shown in Figure 18. As shown, our protocol (especially with high values of ϵ) strongly outperforms the other ones. Specifically, the maximum packet forwarding ratio caused by BSMH for all settings of ϵ is smaller than those caused by LVGR, EDGR and GPSR in all topologies excepting topology 6 in 1-n communication. EDGR and LVGR attain the second-best and third-best performances in most of the cases, and GPSR shows the highest maximum packet forwarding due to the perimeter routing nature. With ϵ=1.2, BSMH results in the maximum packet forwarding ratio which is less than 73% that of LVGR, 81% and EDGR and 64% that of GPSR, in all 1-n communication topologies. In the best cases, the maximum packet forwarding ratio caused by BSMH is smaller 41%, 32% and 27% those of LVGR, EDGR and GPSR, respectively. Regarding the n-n communication, BSMH with ϵ reduces the maximum packet forwarding ratio by the factors of 0.51, 0.52 and 0.44 compared to LVGR, EDGR, and GPSR, respectively. In the best cases, the maximum packet forwarding ratio resulted by BSMH is smaller than 0.18, 0.16 and 0.17 times those of LVGR, EDGR, and GPSR, respectively. The maximum packet forwarding ratio caused by BSMH with ϵ=0.5 is always smaller than 96% that of LVGR, 68% that of EDGR (in 5/6 cases), and 84% that of GPSR in the 1-n communication. With respect to the n-n communication, the maximum packet forwarding ratio caused by BSMH with ϵ=0.5 is always smaller than 60% that of LVGR, 70% that of EDGR and 63% that of GPSR. When reducing ϵ to 0.3, BSMH outperforms all the existing protocols regarding all topologies in the n-n communication and 5/6 cases regarding the 1-n communication.

Regarding Topology 6 in 1-n communication, it can be seen that BSMH with ϵ=0.3 shows the worst performance and BSMH with ϵ=0.5 shows the third-worst performance among all the protocols. This phenomenon can be explained as follows. First, as this topology consists of many small holes, the diversity of routing paths determined by the other protocols (i.e., LVGR, EDGR, GPSR) is increased. Therefore, the performance attained by the other protocols in this topology is better than those attained by the same protocols in other topologies. Secondly, as the spaces between the holes in this topology are small, the scale factors used in the homothetic transformations for determining the Euclidean routing paths in BSMH is limited. In other words, the diversity of routing paths determined by BSMH is degraded in Topology 6. Consequently, BSMH with small values of ϵ cannot improve load balance in comparison with other protocols.

Another observation is that the maximum packet forwarding ratios of all protocols in n-n communication are smaller than those in 1-n communication. This can be seen by comparing Figure 18a,b, where the values plotted in Figure 18b are much smaller than those plotted in Figure 18a. Moreover, the performance gaps between BSMH and the other protocols regarding n-n communication is larger than those regarding 1-n communication. This phenomenon can be explained by the so-called hotspot problem caused by the high traffic load around the destination.

**Figure 19:**
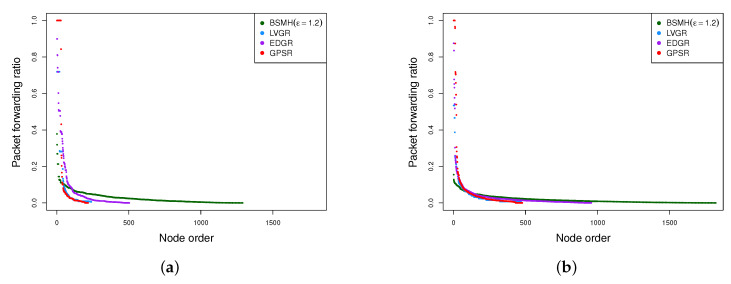
Packet forwarding ratio distribution regarding Topology 4. The x-axis represents the node orders that are sorted by the descending order of the packet forwarding ratio. The y-axis represents the packet forwarding ratios and the x-axis represent the orders of the nodes. (**a**) 1-n communication. (**b**) n-n communication.

To facilitate the understanding, we also plot the distribution of packet forwarding ratios of all nodes in Figure 19. It can be seen that the span of the distributions caused by BSMH in both 1-n and n-n communication are much larger than those caused by the other protocols. This result means that the number of sensors participating in data forwarding in BSMH is much more than those in the other protocols. Moreover, the height of the distribution attained by BSMH is much smaller than those of the other protocols. This result implies that the maximum number of packets forwarded by a node in BSMH is much smaller than those in the other protocols.

#### 5.4.7. Network Lifetime

**Figure 20:**
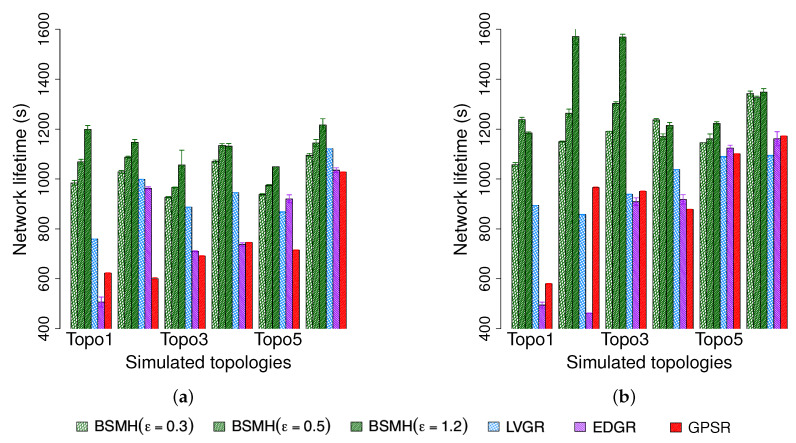
The network lifetime [38]. (**a**) 1-n communication. (**b**) n-n communication.

The network lifetime achieved by the protocols are shown in Figure 20. As shown, the network lifetime achieved by BSMH (especially with high values of ϵ) is much higher than that of the other protocols. LVGR attains the second-best performance in most of the cases. GPSR and EDGR show the smallest network lifetime. With ϵ=1.2, BSMH results in the network lifetime which is higher than 1.1 times that of LVGR, 1.1 times that of EDGR and 1.2 times that of GPSR in 1-n communication, for all network topologies. Specifically, in the best cases, BSMH with ϵ=1.2 can extend the network lifetime by the factors of 1.5, 2.4 an 1.9 compared to LVGR, EDGR and GPSR. Regarding the n-n communication, BSMH with ϵ=1.2 achieved the network lifetime higher than 1.1 times those of LVGR, EDGR and GPSR in all topologies. In the best cases, the ratios of the network lifetime achieved by BSMH to those achieved by LVGR, EDGR and GPSR are 1.8, 3.4 and 2.0, respectively.

Generally, the decrease of ϵ results in the decrease of the network lifetime of BSMH. When reducing ϵ to 0.3, the network lifetime attained by BSMH is still higher than LVGR, EDGR and GPSR in all topologies excepting Topology 6 in 1-n communication. Specifically, in the best cases, the ratios of the network lifetime of BSMH with ϵ=0.3 to those of LVGR, EDGR and GPSR are 1.3, 1.9 and 1.7 in the 1-n communication, and 1.3, 2.5 and 1.8 in the n-n communication, respectively.

Regarding Topology 6 in 1-n communication, it can be seen that BSMH with ϵ=0.3 attains a worse performance than LVGR. This phenomenon can be explained similarly as in Section 5.4.6, i.e., the diversity of routing paths determined by LVGR is increased thanks to the presence of many small holes, while the diversity of routing paths determined by BSMH is reduced due to the small spaces between the holes.

Another observation is that the network lifetimes achieved by 1-n communications are much smaller than those achieved by n-n communications. This can be seen by comparing Figure 20a and Figure 20b. This phenomenon can be explained by the so-called hotspot problem caused by the high traffic load around the destination.

## 6. Conclusions

In this paper, we addressed the network lifetime maximization in wireless sensor networks with the occurrence of multiple holes. As a solution, we have proposed a novel geographic routing protocol named BSMH (i.e., load-Balanced and constant Stretch protocol for bypassing Multiple Holes) for the WSNs. The distinctive feature of BSMH is that its design jointly and concurrently considers three essential network lifetime impacted factors: routing path length, control overhead, and load balancing. As a result, BSMH is considerably a path-length efficient, low-overhead, load-balanced routing protocol for maximum network lifetime in WSNs. We have not only theoretically proven the efficiency of BSMH but also compared to the state-of-the-art protocols by using simulation. The comparative results confirm the superiority of the proposed protocol over the others. Specifically, our proposed protocol extends the network lifetime more than 30% compared to the existing protocols while guaranteeing that the routing path stretch is upper bounded by 1.3. Concerning the setup overhead, BSMH reduces more than 53% compared to a relevant protocol.

The limitation of the protocol is that it requires the location information of the sensors as other geographic routing protocols. Moreover, although our protocol has reduced the setup overhead significantly, it still suffers from extra cost caused by the first two phases (i.e., hole determination and hole information dissemination). Our future works will be devoted to improving these two limitations. Moreover, we will study other realistic impacts such as the transmission range and radio technology on the performance of our proposed protocol.

## Figures and Tables

**Table 1:** Parameters of a sensor node.

Factor	Value
MAC type	CSMA/CA
Interface queue model	DropTail
Transmission of radio	TwoRayGround
Antenna type	OmniAntenna
Queue length	50 packets
Transmission range	40 m
Node initial energy	30 J
Node idle power	9.6 mW
Node receive power	45 mW
Node transmit power	88.5 mW
Packet sending interval	10 s
Data packet size	50 bytes
